# Genetic and environmental influences on structural brain development from childhood to adolescence: A longitudinal twin study on cortical thickness, surface area, and subcortical volume

**DOI:** 10.1016/j.dcn.2024.101407

**Published:** 2024-06-11

**Authors:** L. van Drunen, S. Dobbelaar, E.A. Crone, L.M. Wierenga

**Affiliations:** a Leiden Consortium of Individual Development (L-CID), the Netherlands; b Erasmus University Rotterdam, Social and Behavioral Sciences, the Netherlands; c Leiden Institute for Brain and Cognition (LIBC), the Netherlands; d Institute of Psychology, Leiden University, the Netherlands

**Keywords:** Twin modeling, Structural brain development, Cortical thickness, Surface area, Subcortical volume, Childhood, Early adolescence

## Abstract

The human brain undergoes structural development from childhood to adolescence, with specific regions in the sensorimotor, social, and affective networks continuing to grow into adulthood. While genetic and environmental factors contribute to individual differences in these brain trajectories, the extent remains understudied. Our longitudinal study, utilizing up to three biennial MRI scans (n=485), aimed to assess the genetic and environmental effects on brain structure (age 7) and development (ages 7–14) in these regions. Heritability estimates varied across brain regions, with all regions showing genetic influence (ranging from 18 % to 59 %) with additional shared environmental factors affecting the primary motor cortex (30 %), somatosensory cortex (35 %), DLPFC (5 %), TPJ (17 %), STS (17 %), precuneus (10 %), hippocampus (22 %), amygdala (5 %), and nucleus accumbens (10 %). Surface area was more genetically driven (38 %) than cortical thickness (14 %). Longitudinal brain changes were primarily driven by genetics (ranging from 1 % to 29 %), though shared environment factors (additionally) influenced the somatosensory cortex (11 %), DLPFC (7 %), cerebellum (28 %), TPJ (16 %), STS (20 %), and hippocampus (17 %). These findings highlight the importance of further investigating brain-behavior associations and the influence of enriched and deprived environments from childhood to adolescence. Ultimately, our study can provide insights for interventions aimed at supporting children's development.

## 1 Introduction

The human brain shows structural growth between childhood and adolescence. However, substantial individual differences in developmental patterns have been reported, but it is not well understood what drives these individual differences in growth trajectories (Foulkes and Blakemore, 2018, Mills et al., 2021). These developmental brain changes are a product of environmental and biological factors (Brouwer et al., 2017, Ferschmann et al., 2022, Grasby et al., 2020, Teeuw et al., 2019, Tooley et al., 2021, van Drunen et al., 2023). For example, it was shown that growing up in a low socio-economic status (SES) environment is associated with accelerated brain development (Tooley et al., 2021), and genetic factors associated with mental health problems (e.g., schizophrenia) have been related to reductions in brain volume (Brans et al., 2008). Moreover, there is regional variation in brain developmental trajectories. Particularly brain networks that are associated with sensorimotor, social, and cognitive learning have the most protracted developmental trajectories that continue into adolescence (Mills et al., 2014, Sanders et al., 2022, Tamnes et al., 2017). Interestingly, the period between middle childhood and adolescence is marked by increased social experiences with peers (Crone and Dahl, 2012, Crone and Fuligni, 2020) and by rapid learning of cognitive and sensorimotor skills (Altenmüller and Furuya, 2016, Crone, 2009, Drewing et al., 2006, Germine et al., 2011, Lakhani et al., 2016, Luna et al., 2015, Taubert et al., 2011). Therefore, transitioning from childhood to adolescence may possibly mark an extended period of brain plasticity, that can be influenced by both genes and environment. Yet, it is still unclear to what extent developmental trajectories from childhood to adolescence are driven by genetic and environmental influences. The goal of the present longitudinal twin study was to examine to what extent brain developmental trajectories of sensorimotor, social, and affective brain regions, that were previously shown to follow protracted developmental patterns (Mills et al., 2014, Sanders et al., 2022, Tamnes et al., 2017), are influenced by genetic and/or environmental factors in the transitional period from middle childhood to adolescence.

There are substantial individual differences in the timing of development in cortical and subcortical brain structures. Mills et al. (2021) for example showed that the amount of between-subject variability changes across development. These results indicate that there are differences in underlying driving factors (e.g., genetic input or experiences) on brain development. Yet, little is known on the origins of these individual differences (Foulkes and Blakemore, 2018, Mills et al., 2021). In addition, the developmental patterns differ regionally and by morphological measure. Prior work on regional differences in developmental trajectories of subcortical brain structures reported that some brain structures show increases until early adulthood (e.g., hippocampus) whereas other structures show decreases during early childhood (e.g., nucleus accumbens) (Østby et al., 2009, Tamnes et al., 2017, Wierenga et al., 2014, Wierenga et al., 2018). Additional work on morphological measure differences in brain trajectories reported that cortical thickness shows an early increase during infancy, followed by a subsequent decrease from childhood into early adulthood, whereas surface area shows an early increase continuing into late childhood and a subsequent decrease into adulthood (Aubert-Broche et al., 2013, Mills et al., 2016, Tamnes et al., 2017, Wierenga et al., 2014). It is currently unknown whether relatively protracted developmental patterns are indicative of extended periods of neural plasticity in these regions, and herewith more susceptible to factors such as skill learning.

The differential genetic and environmental effects on brain structure have been assessed using twin designs. These have been extensively studied in adults using cross-sectional samples. Studies showed that the majority of structural brain measures is to a large extent influenced by genetic factors (60–80 %), yet there is substantial regional heterogeneity in heritability estimates (Jansen et al., 2015, Lenroot et al., 2009, Panizzon et al., 2009, Peper et al., 2009, Schmitt et al., 2007, van Soelen et al., 2012, Yoon et al., 2010, Yoon et al., 2011). There are only several studies that included children. One of these was a cross-sectional study in 7–9-year-old children in the same sample that we used in the present study, which showed that regions within the social brain network (temporoparietal junction; TPJ, medial prefrontal cortex; mPFC, superior temporal sulcus; STS, and precuneus) showed large genetic contributions for surface area and cortical thickness. The TPJ, however, showed additional shared environmental influences (van der Meulen et al., 2020). Other longitudinal studies measuring 9 and 12-year-old-children by Swagerman et al. (2014) and 10 and 15 year-year-old-children in Brouwer et al. (2017) showed that baseline levels of subcortical volumes of the affective brain network (i.e., thalamus, hippocampus, amygdala, pallidum, and nucleus accumbens) at age 9, 10, 12, and 15 were all affected by a moderate extent of genetic factors. Again, there was significant heterogeneity in contributing factors where additional shared environmental influences on hippocampus, amygdala, and nucleus accumbens were observed (Brouwer et al., 2017, Swagerman et al., 2014). The cognitive and sensorimotor network was previously found to be more heritable in speech and language related regions in adults (mean age = 48 years old) including regions of Broca and Wernicke, compared to other sensorimotor regions that showed additional shared environmental influences (Thompson et al., 2001). This prior study is based on results in adults, but no study has yet investigated genetic and environmental effects on sensorimotor regions in children.

To what extent the development of brain structure is under genetical or environmental constrains can be examined using longitudinal study designs. There are only few studies that have been able to do so, showing heritable contribution on cortical thickness developmental changes in children (Brouwer et al., 2017, Teeuw et al., 2019; van Soelen, Brouwer, van Baal, et al., 2012) and additional environmental effects on subcortical volume development (Brouwer et al., 2017, Swagerman et al., 2014). Genetic influences on cortical thinning in 9–12-year-olds were prominent in superior and middle frontal areas, superior temporal areas, cingulate, sensorimotor cortices, primary visual, and lateral occipital cortices (ranging from 34 % to 60 %) (van Soelen, Brouwer, van Baal, et al., 2012). However, the study solely assessed genetic contributions to cortical thickness without comparing them to environmental factors. Additionally, shared environment played a more substantial role in subcortical volume development of the thalamus, hippocampus, and amygdala compared to other subcortical regions in 9–15-year-old children (7–23 %) (Brouwer et al., 2017). Heritability estimates of change rates in subcortical volume increased with age, suggesting a growing genetic influence in adults, while environmental factors played a role in children's developmental volumetric differences (Brouwer et al., 2017). Yet, whether the differential morphologies of the cortex show differences in genetic and environmental rates on brain development is currently unknown, although some studies suggested larger genetic input on surface area compared to cortical thickness based on studies in adults and cross-sectional data (Eyler et al., 2012, Panizzon et al., 2009, Winkler et al., 2010). Thus, genetic and environmental influences on the development of cortical thickness, surface area, and subcortical volume of specific regions that show protracted brain development (e.g., the sensorimotor, social, and affective and brain networks) are still understudied and need further investigation.

The present preregistered study included a longitudinal twin sample using up to three biennial MRI assessments of 7–14-year-olds. We first assessed whether brain regions in the sensorimotor, social, and affective network vary in heritability estimates of brain structure in middle childhood (i.e., intercepts). We hypothesized that structure of these brain regions show a moderate to high influence of genetic factors, but the spatio-temporal component must be taken into account since heritability estimates are dependent on location and can change with age (Jansen et al., 2015, Lenroot et al., 2009). Therefore, we expected mostly genetic effects on brain regions in the social, affective and sensorimotor networks but regional heterogeneity in the variances explained by shared environmental input (Swagerman et al., 2014, Thompson-Schill et al., 2005, van der Meulen et al., 2020). Furthermore, we hypothesized a larger genetic contribution to surface area relative to cortical thickness measures (Eyler et al., 2012, Panizzon et al., 2009, Winkler et al., 2010). This hypothesis was based on adult samples and still needs to be investigated in children. For subcortical volumetric regions, we expected to observe contributions of both genetic and shared environmental factors (Swagerman et al., 2014).

In our second aim, we assessed to what extent individual differences in developmental trajectories (i.e., slopes) of cortical thickness, surface area, and subcortical volumetrics of regions of interest (ROIs) are driven by genetic factors, shared environment, and unique environment/measurement error. Developmental changes of regions in the sensorimotor network are expected to be primarily affected by genetic factors based on van Soelen et al. (2012), but they exclusively focused on genetic contributions, precluding environmental comparisons. Development of subcortical regions within the affective network were expected to be (additionally) affected by shared environmental factors (Brouwer et al., 2017, Swagerman et al., 2014). No hypotheses on heritability estimates on development of social brain regions could be made due to the lack of information in prior studies. We expected that development of surface area is to a larger extent driven by genetic factors based on cross-sectional studies (Eyler et al., 2012, Panizzon et al., 2009, Winkler et al., 2010). Yet, the relative contributions of genetic and environmental input on developmental changes of various dimensions of brain structures and brain regions are rarely compared within one longitudinal study. We did not expect differences between the right and left hemispheres and there is insufficient prior literature to formulate hypotheses regarding hemispheric differences in the extent of genetic and environmental influences. Our incorporation of hemispheric analyses was intended to provide a complete examination of genetic and environmental effects. Taken together, this informed us on the regional, dimensional, within- and-between subject-dependent heritability estimates of protracted developmental brain structures.

## 2 Methods

### 2.1 Procedure and participants

The present project was part of the middle childhood cohort (aged 7–14 years) of the longitudinal Leiden Consortium on Individual Development (L-CID) twin study (Crone et al., 2020) that was approved by the Dutch Central Committee on Research Involving Human Subjects (CCMO). Children that took part in the study were same-sex twins born between 2006 and 2009. The zygosity of the twins was confirmed by DNA analyses using cell samples with mouth swabs (Whatman Sterile Omni Swab). The families were recruited via municipal registries and were all living in the western part of the Netherlands (Euser et al., 2016). Children participated in up to three biennial MRI assessments and were included in the study using the following inclusion criteria; twin pairs spoke fluently Dutch, described no psychiatric and neurological impairments, showed normal or corrected-to-normal vision, and had a shared environment at home (i.e., both twins lived in one household 100 % of the time). All MRI assessments took part in the Leiden University Medical Center (LUMC) where both the twin pair and primary parent, described as the parent that spends most time with the twins, were invited for the laboratory visit. Before participation, both parents signed the informed consent. When a child turned 12 years old, both children and parents signed an informed consent.

The MRI sample at the first wave of data collection (Crone et al., 2020) consisted of 418 (of 489) participants (7–9 years old; 46 % boys) that passed inclusion criteria (e.g., manual quality control using Qoala-T procedure Klapwijk et al., 2019). The MRI sample at the second timepoint (wave 3) included 367 (of 409) participants (9–11 years old; 49 % boys). At timepoint 3 (wave 5), the MRI sample included 228 (of 236) participants (11–14 years old; 51 % boys). Over the three MRI timepoints, a total of 485 participants completed one (N=118), two (N=206), or 3 (N=161) MRI assessment(s). See Table 1 for the demographic characteristics. If the scan quality was poor (e.g., excessive movement causing motion rings on T1-images) or technical issues in FreeSurfer prevented analysis of accurately labeling of brain tissue, scans were excluded from further analyses. In our dataset, all technical issues in FreeSurfer's pipeline were related to excessive motion, as determined by manual inspection of these scans. We asked both caregivers of the participants for their education levels in wave 1 (Crone et al., 2020) as a measure of parental education (PE; low, middle high) which is used as a proxy of socio-economic status (SES) in this study. Low levels of PE indicated that both parents completed vocational education at most. Furthermore, high levels of PE indicated that both parents completed at least preparatory college education. The remainder of the combinations of education levels of both parents were included in the middle PE group.

**Table 1:** Demographic characteristics.

	**MRI sample T1 (wave 1)**	**MRI sample T2 (wave 3)**	**MRI sample T3 (wave 5)**	**Heritability sample intercept**	**Heritability sample slope**
*N*	418	367	228	466	320
Boys	46 %	49 %	51 %	48 %	47 %
Left-handed	10 %	12 %	14 %	12 %	13 %
Age (*SD*)	7.97 (.67)	10.01 (.68)	12.28 (.74)	9.68 (1.80)	9.92 (1.91)
Age-range	7.02–9.68	8.97–11.67	11.15–14.11	7.02–14.11	7.02–14.11
Complete twin pairs	179	162	95	233	160
Monozygotic	54 %	53 %	52 %	53 %	53 %
PE: low, middle, high (%)^a^	8.9–43.1-47.6	7.1–41.7-50.7	5.2–44.6-48.1	7.4–43.0-48.9	6.3–42.5-50.7
Median IQ^b^	105	102.5	105	105	105
IQ-range	72.5–137.5	75.0–137.5	75.0–137.5	75.0 137.5	75.0 137.5

*Note*. a Parental education at T1. b Intelligence quotient, based on subtests “similarities” and “block design” of the WISC (3rd edition) at T1.

### 2.2 MRI data acquisition

Before MRI scans were acquired, participants practiced in a mock scanner session to get more comfortable with the scan protocol. This additionally showed to reduce motion during the MRI scan (Achterberg and van der Meulen, 2019, Durston et al., 2009). The MRI scan was acquired on a Philips Ingenia 3.0 Tesla MRI system at LUMC. This MRI scanner included a standard whole-head coil. To further reduce head motion, foam inserts were added next to the ears of the participants. For each participant, a high-resolution 3D T1-weighted scan was acquired using the following MRI scanner settings; voxel size 0.875 × 0.875 x 0.875 mm; TR = 9.72 ms; TE = 4.95 ms; FA = 8°; FOV = 224 (ap) x 177 (rl) x 168 (fh); 140 slices. During the T1-weigthed scan of approximately 5 minutes, participants watched a movie to reduce the possibility of head motion (Greene et al., 2018). Participants were able to watch the movie on a screen located at the end of the magnet bore by looking in a mirror that was attached to the head coil. After the acquisition, the scan was observed for excessive motion (e.g., visible movement rings). The scan was repeated as part of the scan quality protocol in case of excessive motion.

### 2.3 MRI data processing and quality control

The validated software package FreeSurfer (https://surfer.nmr.mgh.harvard.edu/) (v7.1.1), that enables regional brain labeling and tissue classification, was used to process the high-resolution T1-weighted scans. First, a number of automated processing steps were included in the pipeline, such as gray matter segmentation (Fischl et al., 2004, Fischl et al., 2004, Hutton et al., 2009), elimination of non-brain tissue (Clarkson et al., 2011), modification of gray-white matter borders (Ségonne et al., 2007), and intensity normalization (Sled et al., 1998). Next, a longitudinal processing pipeline step was included (Reuter et al., 2012, Reuter and Fischl, 2011) by constructing an unbiased within-subject template, using inverse, robust and consistent registration (Reuter et al., 2010), to improve statistical power and decrease within-subject T1-weighted scan variability.

After the automated and longitudinal processing steps in FreeSurfer, the T1-weigthed scans were all also manually checked for quality by using the Qoala-T procedure published in Klapwijk et al. (2019). As such, the cortical reconstruction of each scan in FreeSurfer (v7.1.1) was manual assessed for excessive head movement, incorrect elimination of non-brain tissue, and missing brain regions. In total, three trained raters performed the manual quality control procedure, such that each scan was rated by two independent raters. For more details on the set of criteria to determine whether cortical reconstruction was sufficient see Klapwijk et al. (2019).

### 2.4 Regions of Interest analyses

Based on prior studies showing that the period between middle childhood and adolescence is important for social development (Crone and Dahl, 2012, Crone and Fuligni, 2020, van der Meulen et al., 2020) and the learning of cognitive and sensorimotor skills (Altenmüller and Furuya, 2016, Crone, 2009, Drewing et al., 2006, Germine et al., 2011, Lakhani et al., 2016, Luna et al., 2015, Taubert et al., 2011), brain regions of interest (ROIs) were involved in the social, affective and sensorimotor brain networks. The mPFC, TPJ, STS, and precuneus play critical roles in the social brain network and are influenced at least partially by environmental factors (Becht et al., 2021, Blakemore, 2008, Crone and Dahl, 2012, Crone and Fuligni, 2020, Mills et al., 2014, van der Meulen et al., 2020, van der Meulen et al., 2023). Therefore, these regions were selected as ROIs for the social network in the current study. Cognitive and sensorimotor skill learning was associated with sensorimotor brain regions, particularly the DLPFC, sensorimotor cortex, cerebellum, and primary motor cortex, which are often implicated in sensorimotor functioning (Altenmüller and Furuya, 2016, Drewing et al., 2006, Lakhani et al., 2016, Luna et al., 2015, Molinari et al., 2007, Patel et al., 2013, Raju and Tadi, 2020, Sanes and Donoghue, 2000, Taubert et al., 2011). Regarding the affective (e.g., emotional) brain network, subcortical ROIs selected for the present study included the hippocampus, amygdala, and nucleus accumbens. These regions were chosen due to their importance in emotional processing (Dieterich et al., 2021, Ibrahim et al., 2024, Phelps, 2006) and their susceptibility to environmental-driven plasticity (Brouwer et al., 2017, Hanson et al., 2015, Kim and Diamond, 2002, Swagerman et al., 2014, van Drunen et al., 2023).

**Fig. 1:**
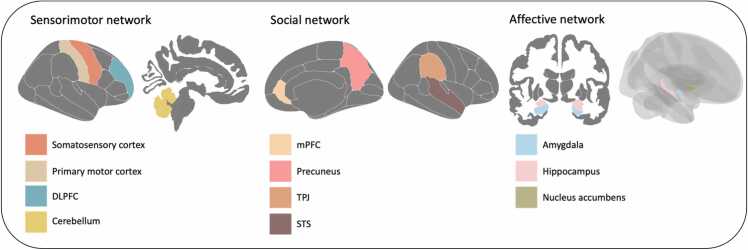
An overview of the ROIs in the sensorimotor, social, and affective brain network. DLPFC = dorsolateral prefrontal cortex, mPFC = medial prefrontal cortex, TP*J* = temporoparietal junction, STS = superior temporal sulcus.

The ROIs were based on atlases in FreeSurfer (aseg (Fischl et al., 2002) and Desikan-Killiany (DK; Desikan et al., 2006)). Both CT and SA are reported for cortical ROIs, whereas for subcortical measures we included volumetrics. These include the following right and left regions of the social brain network: mPFC (rostral anterior cingulate in DK atlas), TPJ (supra marginal in DK atlas), STS (superior temporal in aseg atlas) and precuneus (DK atlas). The following left and right brain regions of the sensorimotor network were selected: dorsolateral prefrontal cortex (DLPFC; rostral middle frontal in DK atlas), cortical sensorimotor region (postcentral in DK atlas), cerebellum (aseg atlas), and primary motor cortex (precentral in DK atlas). And finally, the following right and left subcortical regions of the affective network were selected: hippocampus (aseg atlas), amygdala (aseg atlas), and nucleus accumbens (aseg atlas). See Fig. 1 for an overview of all ROIs. We included 11 brain regions in total.

To correct for multiple testing, we used Sidak adjustment on our linear mixed model analyses corrected for correlated variables (e.g., for cortical thickness, surface area, and subcortical volume; http://www.quantitativeskills.com/sisa/calculations/bonfer.htm). We did so by computing Pearson correlations between all regions (including right and left hemispheres) within a single morphological dimension, such as for cortical thickness, surface area, and subcortical volume separately. The average correlation between ROIs was 0.26 for cortical thickness, 0.28 for surface area, and 0.42 for subcortical volume. This resulted in an adjusted significant threshold of 0.016 (n=14 tests) for cortical thickness, 0.017 (n=14 tests) for surface area, and 0.025 (n=8 tests) for subcortical volume.

### 2.5 Linear mixed-effects model analyses

Before we test our hypotheses, we first assessed whether there were group level age related changes in the ROIs of the sensorimotor, social, and affective network. To investigate whether there were significant brain developmental group changes of ROIs, we used linear mixed-effects models that were performed using the lme4 package (Bates et al., 2014) in R (R Core Team, 2015). We inspected the results with the type III ANOVA’s Satterthwaite’s method. If significant main effects were observed, we moved on to post hoc testing using least-square means with Kenward–Roger corrected degrees of freedom and Sidak-adjusted *p*-values. In the linear-mixed models, the effects of age and sex (male, female: assessed trough parent report) on the ROI were assessed. The intercepts were estimated at the minimum age of the sample (age minimum = 7.02 years). Random intercepts of the child (i) and family (j) were included to account for the nesting effects between twin pairs and within families (ChildID and FamilyID). We specified the fitted linear mixed-effects model in R as:

$$\sum_{i=1}^{N}Y_{\mathrm{ijk}}\;∼\;\beta_{1}\left({\mathrm{age}}_{\mathrm{ik}}\right)+\beta_{2}\left({\mathrm{sex}}_{i}\right)+\beta_{3}\left({\mathrm{age}}_{\mathrm{ik}}*{\mathrm{sex}}_{i}\right)+\beta_{4}\left({\mathrm{zygosity}}_{i}\right)+\;\beta_{0\mathrm{ij}}\;+\;{e^{s}}_{\mathrm{ij}}\;+\;\varepsilon_{\mathrm{ijk}}$$

Here, $Y_{\mathrm{ijk}}$ reflects the brain measures of child i where ${\mathrm{age}}_{\mathrm{ik}}$ is the age of child i at timepoint k minus the minimal age of the sample including child’s sex ${\mathrm{sex}}_{i}$ and ${\mathrm{zygosity}}_{i}$. In addition, two random effects were included, where $\beta_{0\mathrm{ij}}$ reﬂects the individual’s random intercept nested within family and ${e^{s}}_{\mathrm{ij}}$ includes their random slope.

To estimate individual level brain structure and growth patterns, we used intercepts and slopes estimates from subject based linear models. Herewith, we deviated from our initial preregistered approach by extracting brain growth parameters for each ROI using a linear age model per individual, following prior work (Blankenstein et al., 2020, Pfister et al., 2013, van der Cruijsen et al., 2023). For each individual, intercepts and slopes were estimated by using the lm function in R (R Core Team, 2015). To this end, from each linear model the regression intercept (i.e., individual intercept: brain structure at 7.02 years of age) and regression coefficient (i.e., individual slope: brain development between 7 and 14 years of age) were saved for each ROI and used for subsequent analyses where we investigated genetic modeling. For the individual slope estimation, we solely included participants with two and three timepoints of brain data (N=367). For the intercept estimation, we included participants with at least one MRI wave of brain data (n=485).

### 2.6 Genetic modeling

First, we investigated the genetic and environmental influences on the intercepts (at age 7.02) of cortical thickness, surface area, and subcortical volume of brain regions in the social, affective, and sensorimotor network in middle childhood. Second, we investigated the genetic and environmental influences on the slopes (age 7–14) of cortical thickness, surface area and subcortical volume of our ROIs. To do so, we first performed within-twin pair Pearson correlations for each ROI, based on the complete MRI samples (N-intercept = 485; N-slope = 367) (Achterberg et al., 2018, van der Meulen et al., 2018, van Drunen et al., 2021).

As a subsequent step, we used structural equation ACE modeling to explain the variation of brain structure and development of ROIs by additive genetic (A), common shared environment (C), and unique environment/measurement error (E) driven influences. We did so by using the OpenMX package (Neale et al., 2016; version 2.7.4) in R (R Core Team, 2015). Because all twin pairs have a shared environment at home, the within-twin correlation of the shared environmental factor in the model was set to 1 for MZ and DZ twins. Additionally, the within-twin pair correlation of the genetic factor was set to 0.5 for DZ twins since they share approximately 50 % of their genes and set to 1 for MZ twins as they share 100 % of their genes. We included free estimates for the within-twin correlations of the unique environment/measurement error effect in the model (Neale et al., 2016).

We reported the heritability estimates using the following steps: 1) We reported on higher genetic input (A) whenever MZ within-twin pair correlations were significantly higher than DZ within-twin pair correlations. 2) We interpreted an effect as additional common shared environmental input (C) whenever MZ and DZ twin-pair correlations were both significant but not significantly different. 3) We reported on the contribution of genetic (A), common shared environment (C) and unique environment/measurement error (E) whenever the confidence interval includes no zero. To observe patterns in genetic and environmental influences on brain networks (sensorimotor, social, and affective), ROIs, dimensions (cortical thickness, surface area, and volume) and intercept/slope, we conducted a descriptive comparison of heritability estimates between the different regional networks. We calculated mean scores of the reported percentages of the contributions of additive genetic (A), common shared environment (C) and unique environment/measurement error (E) for brain structure and development across networks, ROIs, dimensions, and dimensions per network.

## 3 Results

### 3.1 Age effects

To assess whether there were significant group level age-related changes in our ROIs we tested thirty-six linear mixed models. We determined with these analyses whether cortical thickness and surface area of the primary motor cortex, somatosensory cortex, DLPFC, mPFC TPJ, STS, precuneus, and subcortical volume of cerebellum, hippocampus, amygdala, and nucleus accumbens developed over time. The main and interaction effects of age, sex, and zygosity are reported in Table S1-A and B of the supplementary materials. The parameter estimates of intercepts and slopes of cortical thickness, surface area, and volume of the ROIs are displayed in Table 2-A, Table 2-B. Furthermore, see Fig. 1 for an overview of the ROIs. As can be seen in Table 2, there were significant age-related changes in all ROIs between 7 and 14 years old, except for TPJ surface area. Cortical thickness predominantly showed age-related decreases in the brain regions of the sensorimotor and social networks, only cortical thickness in the primary motor cortex showed an age-related increase. Surface area showed age-related decreases in the somatosensory cortex and precuneus, whereas increases in the primary motor cortex, DLPFC, mPFC, and STS. Finally, subcortical volume showed age-related increases in the cerebellum, amygdala, and right nucleus accumbens. Volumetric decreases were observed in the hippocampus and right nucleus accumbens. Visualizations of age-related brain developmental trajectories of all ROIs can be seen in Figure S1 of the supplementary materials.

**Table 2-A:** Parameter estimates of intercepts and slopes of cortical thickness and surface area of cortical ROIs.

		**Primary motor**		**Somatosensory**		**DLPFC**		**mPFC**		**TPJ**		**STS**		**Precuneus**	
		**Right**	**Left**	**Right**	**Left**	**Right**	**Left**	**Right**	**Left**	**Right**	**Left**	**Right**	**Left**	**Right**	**Left**
**Cortical thickness**															
	Intercept (SE)	2.75^***^ (.02)	2.81^***^ (.01)	2.41^***^ (.01)	2.43^***^ (.02)	2.77^***^ (.01)	2.85^***^ (.01)	3.30^***^ (.02)	3.30^***^ (.02)	2.95^***^ (.01)	2.96^***^ (.01)	3.20^***^ (.01)	3.16^***^ (.01)	2.86^***^ (.01)	2.85^***^ (.01)
	Slope (SE)	.01^***^ (.002)	.01^***^ (.001)	-.004^**^ (.002)	-.005^**^ (.002)	-.01 (.002)	-.02^***^ (.003)	-.03^***^ (.002)	-.03^***^ (.002)	-.01^***^ (.002)	-.01^***^ (.002)	-.01^***^ (.002)	-.01^***^ (.002)	-.02^***^ (.002)	-.02^***^ (.002)
**Surface area**															
	Intercept (SE)	4591^***^ (48.78)	4594^***^ (47.02)	3947^***^ (42.59)	4077^***^ (44.01)	5309^***^ (83.37)	5097^***^ (73.72)	584^***^ (11.43)	750^***^ (14.46)	3695^***^ (56.58)	4104^***^ (65.99)	3613^***^ (38.96)	3951^***^ (42.64)	3992^***^ (52.75)	3807^***^ (48.16)
	Slope (SE)	18.65^***^ (1.77)	21.63^***^ (2.56)	-8.08^***^ (1.83)	-5.84* (2.27)	79.64^***^ (6.98)	73.57^***^ (7.48)	7.38^***^ (.78)	10.53^***^ (.87)	-2.50 (1.66)	.64 (2.04)	14.17^***^ (1.58)	9.70^***^ (1.86)	-10.67^***^ (1.75)	-9.18^***^ (1.58)

*Note.* DLPFC = dorsolateral prefrontal cortex, mPFC = medial prefrontal cortex, TPJ = temporoparietal junction, STS = superior temporal sulcus. * = significant *p*-value of <.027 (cortical thickness) or <.031 (surface area), ^**^ = significant *p*-value of <.01, ^***^ = significant *p*-value of <.001. Intercept was measured at minimum age of 7.02 years.

**Table 2-B:** Parameter estimates of intercept and slope of subcortical volumetric ROIs.

		**Cerebellum**		**Hippocampus**		**Amygdala**		**Nucleus accumbens**	
		**Right**	**Left**	**Right**	**Left**	**Right**	**Left**	**Right**	**Left**
**Subcortical volume**									
	Intercept (SE)	56365^***^ (509.35)	56500^***^ (496.46)	4360^***^ (41.84)	4259^***^ (39.19)	1758^***^ (19.49)	1563^***^ (24.57)	790^***^ (11.57)	669^***^ (12.10)
	Slope (SE)	515.94^***^ (41.01)	413.33^***^ (40.85)	-14.34^**^ (4.08)	-14.35^***^ (4.08)	9.75^***^ (1.61)	5.56^**^ (1.79)	3.95^**^ (1.26)	-10.24^***^ (1.67)

*Note.* * = significant *p*-value of <.029, ^**^ = significant *p*-value of <.01, ^***^ = significant *p*-value of <.001.

### 3.2 Heritability analyses on brain structure and development

To investigate the contributions of genetic and environmental influences on variances of brain structure in middle childhood and development of ROIs in the social, affective, and sensorimotor network, we first performed within-twin pair Pearson correlations based on the complete MRI sample (See Table 3 for an overview of the within-twin correlations of MZ and DZ twins on the ROIs, including Z-scores indicating the significance of the difference between MZ and DZ correlations.

**Table 3:** Within-twin pair correlations and contributions of ACE genetic modeling for intercept (brain structure in middle childhood) and slope (brain development).

***ROI***		***MZ***	***DZ***	***Z***	***Model***	**A²**	**C²**	**E²**
***Sensorimotor network***								
*Primary motor CT right (i)*	*r*	.17	.12	.40	*ACE*	.15	.03	.82
	*p*	.07	.20		*95 % CI*	[.00-.36]	[.00-.26]	[.65-.98]
*Primary motor CT left (i)*	*r*	.12	.21	-.73	*ACE*	.00	.15	.85
	*p*	.18	<.05		*95 % CI*	[.00-.27]	[.00-.28]	[.71-.98]
*Primary motor CT right (s)*	*r*	.33	.04	2.39**	*ACE*	**.27**	.00	.73
	*p*	<.01	.76		*95 % CI*	**[.00-.44]**	[NA-.26]	[.56-.92]
*Primary motor CT left (s)*	*r*	.04	.13	-.72	*ACE*	.00	.06	.94
	*p*	.72	.28		*95 % CI*	[NA-.23]	[NA-.21]	[.77–1.00]
*Primary motor SA right (i)*	*r*	.50	.36	1.36	*ACE*	**.34**	**.17**	.49
	*p*	<.001	<.001		*95 % CI*	**[.00-.61]**	**[.00-.48]**	[.39-.64]
*Primary motor SA left (i)*	*r*	.37	.50	-1.27	*ACE*	.00	**.43**	.57
	*p*	<.001	<.001		*95 % CI*	[.00-.27]	**[.19-.53]**	[.47-.68]
*Primary motor SA right (s)*	*r*	.14	.06	.64	*ACE*	.14	.00	.86
	*p*	.19	.61		*95 % CI*	[.00-.33]	[NA-.25]	[.67–1.00]
*Primary motor SA left (s)*	*r*	.07	.08	-.08	*ACE*	.00	.09	.91
	*p*	.50	.49		*95 % CI*	[NA-.18]	[.00-.24]	[.76-NA]
*Somatosensory CT right (i)*	*r*	.33	.27	.52	*ACE*	**.20**	**.15**	.65
	*p*	<.001	<.01		*95 % CI*	**[.00-.50]**	**[.00-.40]**	[.50-.81]
*Somatosensory CT left (i)*	*r*	.18	.05	1.04	*ACE*	**.16**	.00	.84
	*p*	<.05	.59		*95 % CI*	**[.00-.30]**	[.00-.24]	[.70–1.00]
*Somatosensory CT right (s)*	*r*	.18	.36	-1.54	*ACE*	.00	.27	.73
	*p*	.10	<.01		*95 % CI*	[NA-37]	[.00-.41]	[.58-.89]
*Somatosensory CT left (s)*	*r*	.07	-.04	.87	*ACE*	.05	.00	.95
	*p*	.51	.73		*95 % CI*	[00-.22]	[NA-.17]	[.77–1.00]
*Somatosensory SA right (i)*	*r*	.57	.29	2.75**	*ACE*	**.52**	**.03**	.45
	*p*	<.001	<.01		*95 % CI*	**[.15-.66]**	**[.00-.36]**	[.34-.58]
*Somatosensory SA left (i)*	*r*	.54	.39	1.52	*ACE*	**.32**	**.22**	.46
	*p*	<.001	<.001		*95 % CI*	**[.00-.64]**	**[.00–51]**	[.39-.59]
*Somatosensory SA right (s)*	*r*	.24	.23	.08	*ACE*	**.03**	**.22**	.75
	*p*	<.05	<.05		*95 % CI*	**[NA-.42]**	**[.06-.36]**	[.61-.91]
*Somatosensory SA left (s)*	***r***	**.31**	**.14**	1.42	*ACE*	**.31**	.00	.69
	***p***	**<.01**	**.22**		*95 % CI*	**[.13-.47]**	[NA-.34]	[.53-.97]
*DLPFC CT right (i)*	*r*	.25	.21	.33	*ACE*	**.06**	**.19**	.75
	*p*	<.01	<.05		*95 % CI*	**[.00-.40]**	**[.00-.35]**	[.60-.89]
*DLPFC CT left (i)*	*r*	.30	.12	1.49*	*ACE*	**.26**	.01	.73
	*p*	<.001	.20		*95 % CI*	**[.00-.41]**	[.00-.32]	[.59-.88]
*DLPFC CT right (s)*	*r*	.15	.26	-.91	*ACE*	.00	**.20**	.80
	*p*	.16	<.05		*95 % CI*	[NA-.33]	**[.00-.34]**	[.65-.96]
*DLPFC CT left (s)*	*r*	.32	.11	1.74*	*ACE*	**.28**	.00	.72
	*p*	<.01	.36		*95 % CI*	**[.00-.44]**	[NA-.33]	[.56-.90]
*DLPFC SA right (i)*	*r*	.39	.16	1.97*	*ACE*	**.36**	.00	.64
	*p*	<.001	.10		*95 % CI*	**[.22-.49]**	[NA-.34]	[.51-.78]
*DLPFC SA left (i)*	*r*	.43	.06	3.15**	*ACE*	**.37**	.00	.63
	*p*	<.001	.56		*95 % CI*	**[.09-.50]**	[.00-.22]	[.50-.78]
*DLPFC SA right (s)*	*r*	.30	.25	.43	*ACE*	**.09**	**.20**	.71
	*p*	<.01	<.05		*95 % CI*	**[.00-.45]**	**[.02-.34]**	[.57-.87]
*DLPFC SA left (s)*	*r*	.31	-<.01	2.52**	*ACE*	**.24**	.00	.76
	*p*	<.01	.94		*95 % CI*	**[.07-.41]**	[NA-.15]	[.60-.93]
*Cerebellum volume right (i)*	*r*	.62	.26	3.62***	*ACE*	**.61**	.00	.39
	*p*	<.001	<.01		*95 % CI*	**[.50-.70]**	[.00-.24]	[.30-.50]
*Cerebellum volume left (i)*	*r*	.56	.26	2.89**	*ACE*	**.56**	.00	.44
	*p*	<.001	<.01		*95 % CI*	**[.44-.67]**	[NA-.27]	[.33-.56]
*Cerebellum volume right (s)*	*r*	.25	.30	-.43	*ACE*	**.04**	**.24**	.72
	*p*	<.05	<.05		*95 % CI*	**[NA-.25]**	**[.12-.41]**	[.59-.88]
*Cerebellum volume left (s)*	*r*	.28	.35	-.61	*ACE*	.00	**.31**	.69
	*p*	<.01	<.01		*95 % CI*	[NA-.46]	**[.16-.44]**	[.56-.84]
***Social network***								
*mPFC CT right (i)*	*r*	.10	.13	-.24	*ACE*	.00	.11	.89
	*p*	.28	.17		*95 % CI*	[.00-.26]	[.00-.23]	[.77–1.00]
*mPFC CT left (i)*	*r*	.25	.11	1.14	*ACE*	**.24**	.00	.76
	*p*	<.01	.24		*95 % CI*	**[.00-.39]**	[.00-.28]	[.61-.93]
*mPFC CT right (s)*	*r*	.001	.002	-.01	*ACE*	.00	.00	1.00
	*p*	.99	.98		*95 % CI*	[NA-.19]	[NA-.15]	[.81-NA]
*mPFC CT left (s)*	*r*	.26	-.001	2.11*	*ACE*	**.22**	.00	.78
	*p*	<.05	.99		*95 % CI*	**[.00-.40]**	[NA-.24]	[.60-.98]
*mPFC SA right (i)*	*r*	.38	.05	2.76**	*ACE*	**.33**	.00	.67
	*p*	<.001	.62		*95 % CI*	**[.06-.47]**	[.00-.20]	[.53-.83]
*mPFC SA left (i)*	*r*	.34	.15	1.61*	*ACE*	**.32**	.00	.69
	*p*	<.001	.13		*95 % CI*	**[.00-.44]**	[.00-.33]	[.56-.85]
*mPFC SA right (s)*	*r*	.05	-.03	.63	*ACE*	.02	.00	.98
	*p*	.67	.82		*95 % CI*	[NA-.21]	[NA-.16]	[.79-NA]
*mPFC SA left (s)*	*r*	.08	-.15	1.82	*ACE*	.01	.00	.99
	*p*	.48	.20		*95 % CI*	[NA-.18]	[NA-.12]	[.82-.NA]
*TPJ CT right (i)*	*r*	.20	.36	-1.37	*ACE*	.00	**.27**	.73
	*p*	<.05	<.001		*95 % CI*	[.00-.30]	**[.02-.39]**	[.61-.85]
*TPJ CT left (i)*	*r*	.10	.17	-.56	*ACE*	.00	.14	.86
	*p*	.27	.08		*95 % CI*	[.00-.29]	[.00-.27]	[.70-.98]
*TPJ CT right (s)*	*r*	.08	.32	-1.98*	*ACE*	.00	**.18**	.82
	*p*	.44	<.01		*95 % CI*	[NA-.30]	**[.00-.33]**	[.67-.96]
*TPJ CT left (s)*	*r*	-.003	.22	-1.79*	*ACE*	.00	**.12**	.88
	*p*	.97	<.05		*95 % CI*	[NA-.29]	**[NA-.27]**	[.70-NA]
*TPJ SA right (i)*	*r*	.51	.40	1.10	*ACE*	**.27**	**.23**	.50
	*p*	<.001	<.001		*95 % CI*	**[.00-.61]**	**[.00-.51]**	[.38-.63]
*TPJ SA left (i)*	*r*	.48	.24	2.20*	*ACE*	**.48**	.00	.52
	*p*	<.001	<.05		*95 % CI*	**[.06-.59]**	[.00-.34]	[.41-.66]
*TPJ SA right (s)*	*r*	.22	.17	.41	*ACE*	.02	**.18**	.80
	*p*	<.05	.14		*95 % CI*	[NA-.38]	**[.04-.34]**	[.65-.95]
*TPJ SA left (s)*	*r*	.02	.001	.15	*ACE*	.01	.00	.99
	*p*	.83	.99		*95 % CI*	[NA-.19]	[NA-.16]	[.81-NA]
*STS CT right (i)*	*r*	.18	.14	.32	*ACE*	.12	.06	.82
	*p*	<.05	.14		*95 % CI*	[.00-.34]	[.00-.28]	[.66-.97]
*STS CT left (i)*	*r*	.27	.21	.50	*ACE*	**.16**	**.12**	.72
	*p*	<.01	<.05		*95 % CI*	**[.00-.44]**	**[.00-.35]**	[.56-.88]
*STS CT right (s)*	*r*	<.001	.01	-.08	*ACE*	.00	.00	1.00
	*p*	.99	.97		*95 % CI*	[NA-.18]	[NA-.15]	[.82-NA]
*STS CT left (s)*	*r*	.02	.13	-.87	*ACE*	.00	.05	.95
	*p*	.85	.28		*95 % CI*	[NA-.21]	[.00-.21]	[.79-NA]
*STS SA right (i)*	*r*	.58	.40	1.88*	*ACE*	**.37**	**.20**	.43
	*p*	<.001	<.001		*95 % CI*	**[.02-.66]**	**[.00-.48]**	[.33-.56]
*STS SA left (i)*	*r*	.54	.36	1.79*	*ACE*	**.36**	**.19**	.45
	*p*	<.001	<.001		*95 % CI*	**[.00-.64]**	**[.00-.49]**	[.35-.58]
*STS SA right (s)*	*r*	.11	0.01	.79	*ACE*	.07	.00	.93
	*p*	.31	.95		*95 % CI*	[NA-.25]	[NA-.19]	[.75-NA]
*STS SA left (s)*	*r*	.17	.22	-.41	*ACE*	.00	**.20**	.80
	*p*	.11	<.05		*95 % CI*	[NA-.37]	**[.05-.34]**	[.66-.95]
*Precuneus CT right (i)*	*r*	.18	.21	-.25	*ACE*	.00	**.18**	.82
	*p*	.05	<.05		*95 % CI*	[.00-.33]	**[.00-.31]**	[.66-.94]
*Precuneus CT left (i)*	*r*	.18	-.01	1.51*	*ACE*	**.15**	.00	.85
	*p*	<.05	.95		*95 % CI*	**[.00-.30]**	[.00-.20]	[.69–1.00]
*Precuneus CT right (s)*	*r*	-.06	-.01	.55	*ACE*	.00	.00	1.00
	*p*	.58	.94		*95 % CI*	[NA-13]	[NA-.11]	[.86-NA]
*Precuneus CT left (s)*	*r*	-<.01	-.28	2.27*	*ACE*	.00	.00	1.00
	*p*	.99	<.05		*95 % CI*	[NA-.11]	[NA-.07]	[.88-NA]
*Precuneus SA right (i)*	*r*	.67	.43	2.77**	*ACE*	**.46**	**.20**	.34
	*p*	<.001	<.001		*95 % CI*	**[.15-.73]**	**[.00-.47]**	[.26-.44]
*Precuneus SA left (i)*	*r*	.65	.31	3.58***	*ACE*	**.64**	.00	.36
	*p*	<.001	<.001		*95 % CI*	**[.33-.72]**	[.00-.27]	[.27-.46]
*Precuneus SA right (s)*	*r*	.32	.08	1.98*	*ACE*	**.29**	.00	.71
	*p*	<.01	.50		*95 % CI*	**[.11-.45]**	[NA-.30]	[.54-.89]
*Precuneus SA left (s)*	*r*	.19	.10	.73	*ACE*	.14	.05	.82
	*p*	.07	.38		*95 % CI*	[.00-.35]	[NA-.30]	[.65-.99]
***Affective network***								
*Hippocampus volume right (i)*	*r*	.52	.35	1.66*	*ACE*	**.25**	**.25**	.50
	*p*	<.001	<.001		*95 % CI*	**[.00-.60]**	**[.00-.52]**	[.39-.64]
*Hippocampus volume left (i)*	*r*	.54	.35	1.88*	*ACE*	**.34**	**.19**	.47
	*p*	<.001	<.001		*95 % CI*	**[.00-.63]**	**[.00-.49]**	[.37-.61]
*Hippocampus volume right (s)*	*r*	.36	.25	.96	*ACE*	**.17**	**.17**	.66
	*p*	<.001	<.05		*95 % CI*	**[.00-.50]**	**[.00-.35]**	[.51-.82]
*Hippocampus volume left (s)*	*r*	.41	.30	.99	*ACE*	**.23**	**.17**	.60
	*p*	<.001	<.01		*95 % CI*	**[.00-.56]**	**[.00-.45]**	[.47-.77]
*Amygdala volume right (i)*	*r*	.46	.28	1.65*	*ACE*	**.41**	**.06**	.53
	*p*	<.001	<.01		*95 % CI*	**[.00-.59]**	**[.00-.39]**	[.41-.68]
*Amygdala volume left (i)*	*r*	.42	.23	1.68*	*ACE*	**.38**	**.04**	.58
	*p*	<.001	<.05		*95 % CI*	**[.00-.54]**	**[.00-.38]**	[.46-.74]
*Amygdala volume right (s)*	*r*	.22	.11	.89	*ACE*	**.22**	.00	.78
	*p*	<.05	.37		*95 % CI*	**[.03-.39]**	[NA-.31]	[.61-.97]
*Amygdala volume left (s)*	*r*	.32	-.01	2.69**	*ACE*	**.26**	.00	.74
	*p*	<.01	.96		*95 % CI*	**[.07-.44]**	[NA-.24]	[.56-.93]
*N. accumbens volume right (i)*	*r*	.46	.05	3.53***	*ACE*	**.38**	.00	.62
	*p*	<.001	.57		*95 % CI*	**[.08-.50]**	[.00-.25]	[.50-.76]
*N. accumbens volume left (i)*	*r*	.22	.19	.25	*ACE*	**.02**	**.20**	.78
	*p*	<.05	<.05		*95 % CI*	**[.00-.37]**	**[.00-.33]**	[.63-.91]
*N. accumbens volume right (s)*	*r*	.31	.08	1.90*	*ACE*	**.26**	.00	.74
	*p*	<.01	.51		*95 % CI*	**[.09-.42]**	[NA-.33]	[.58-.91]
*N. accumbens volume left (s)*	*r*	.11	.04	.56	*ACE*	.11	.00	.89
	*p*	.33	.73		*95 % CI*	[.00-.29]	[NA-.23]	[.71-NA]

*Note*. i = intercept; s = slope; mPFC = medial prefrontal cortex; STS = superior temporal sulcus; TPJ = temporoparietal junction; DLPFC = dorsolateral prefrontal cortex; r = Pearson correlation; Z = test statistic z, significant Z-scores indicate significant difference between MZ and DZ correlations. *p<.05, **p<.01, ***p<.001.

**Fig. 2:**
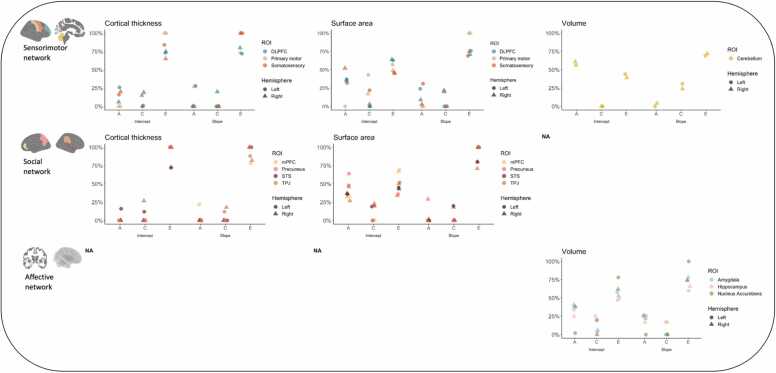
An overview of the ACE contributions on intercepts and slopes of ROIs per network.

As a subsequent step, we used structural equation ACE modeling to explain the variation of brain structure and development of ROIs by additive genetic (A), common shared environment (C), and unique environment/measurement error (E) driven influences. Table 3 reports a compete overview of the relating ACE contributions of cortical thickness, surface area and subcortical volume of intercepts and slopes of the right and left ROIs within the sensorimotor, social, and affective networks including the 95 % confidence intervals (CI). The analyses are organized by brain network (sensorimotor, social, and affective) and separated by dimension (cortical thickness, surface area, and volume) and intercept/slope (intercept: brain structure in middle childhood at age 7.02, slope: structural brain development between 7 and 14 years of age). The reported values in the text are displayed in bold in the table based on the steps described in the Methods section. The bold values are displayed in Fig. 2 with an overview of heritability estimates on the regions within the sensorimotor, social, and affective networks.

As the intercept results are dependent on the set age (youngest participant in our sample), we have, for completeness, tested additional intercept analysis at age 14.11 years (the oldest participant in our dataset). We estimated the structural equation ACE models for variation of brain structure (i) for all ROIs and tested additive genetic (A), common shared environment (C), and unique environment/measurement error (E) driven influences. The values are displayed in Table S2 of the Supplement materials with an overview of heritability estimates on the regions within the sensorimotor, social, and affective networks.

#### 3.2.1 Sensorimotor network

Cortical thickness

*Intercept*

Within the sensorimotor network, variation in cortical thickness of the right somatosensory cortex was accounted for by a combination of genetic and shared environmental input (A=20 % and C=15 %). For DLPFC cortical thickness, variation of right DLPFC thickness was partly driven by a combination of genetic and shared environmental influences (A=6 % and C=19 %) whereas left DLPFC was mainly explained by genetic contributions (A=26 %, C=1 %). Within-twin pair correlations of primary motor (both hemispheres) and left somatosensory cortical thickness in MZ and DZ twins were not significant.

*Slope*

Within the sensorimotor network, ACE modeling indicated that variation in right primary motor thickness slopes was partly explained by genetic factors (A=27 %). In addition, variation in left DLPFC was driven partly by genetic input (A=28 %). The within-twin pair correlations of left primary motor cortex, somatosensory cortex (both hemispheres) and right DLPFC thickness slopes in MZ and DZ twins were not significant.

Surface area

*Intercept*

Within the sensorimotor network, ACE modeling indicated that variation in surface area of the right primary motor cortex was partly accounted for by a combination of genetic factors and shared environment (A=34 % and C=17 %) whereas variation in surface area of the left primary motor cortex was largely driven by shared environment (C=43 %). For surface area of the somatosensory cortex, the right hemisphere was approximately half driven by genetic input (A=52 %, C=3 %) and the left hemisphere by a combination of genetic and shared environmental factors (A=32 % and C=22 %). Variations of surface area in the right and left DLPFC were partly explained by genetic input (right DLPFC: A=36 %, left DLPFC: 37 %).

*Slope*

Within the sensorimotor network, variation in surface area slopes of the right somatosensory cortex was partly driven by shared environment with a relatively minor contribution from genetic factors (C=22 %, A=3 %). Variation in left somatosensory surface area was partly driven by genetic factors (A=31 %). For DLPFC surface area slopes, variation in the right hemisphere was partly driven by a combination of genetic factors and shared environment (A=9 % and C=20 %) whereas variation in the left hemisphere was partly explained by genetic factors (A=24 %). Within-twin pair correlations of primary motor (both hemispheres) surface area slopes in MZ and DZ twins were not significant.

Volume

*Intercept*

Within the sensorimotor network, variation in right and left volumetric cerebellum cortex was mainly explained by genetic factors (right cerebellum: A=61 %, left cerebellum A=56 %).

*Slope*

Within the sensorimotor network, variations in volumetric cerebellum slopes of both hemispheres were partly explained by shared environment (right cerebellum: C=24 %, left cerebellum: C=31 %).

#### 3.2.2 Social network

Cortical thickness

*Intercept*

Within de social network, results indicated that variation in right TPJ thickness was partly accounted for by shared environment (C=27 %). For left STS thickness, the variation was partly explained by a combination of genetic and shared environmental input (A=16 % and C=12 %). Within-twin pair correlations of mPFC (both hemispheres), left TPJ, right STS, and precuneus (both hemispheres) thickness in MZ and DZ twins were not significant.

*Slope*

Within de social network, variation in thickness slopes of the left mPFC was partly driven by genetic factors (A=22 %), whereas variation in right and left TPJ thickness slopes were partly explained by shared environment (right TPJ: C=18 %, left TPJ: C=12 %). The within-twin pair correlations of right mPFC, STS (both hemispheres), and precuneus (both hemispheres) thickness slopes in MZ and DZ twins were not significant or significantly different.

Surface area

*Intercept*

Within de social network, variations of right and left mPFC surface area were partly explained by genetic control (right mPFC: A=33 %, left mPFC: A=32 %). For TPJ surface area, variation of the right hemisphere was half explained by a combination of genetic and shared environmental input (A=27 % and C=23 %) whereas the left hemisphere was half explained by genetic factors (A=48 %). Variation of right precuneus surface area was mainly explained by a combination of genetic factors and shared environment (A=46 % and C=20 %) whereas variation of left precuneus surface area was mainly explained by genetic control (A=64 %).

*Slope*

Within de social network, ACE modeling indicated that variation in surface area slopes of the right TPJ was partly accounted for by shared environment with a minor contribution of genetic factors (C=18 %, A=2 %). Variation in right precuneus surface area slopes was partly explained by genetic control (A=29 %). Within-twin pair correlations of mPFC (both hemispheres), left TPJ, right STS, and left precuneus surface area slopes in MZ and DZ twins were not significant.

#### 3.2.3 Affective network

Volume

*Intercept*

Within de affective network, ACE modeling indicated that variations of right and left volumetric hippocampus were half explained by a combination of genetic and shared environmental input (right hippocampus: A=25 % and C=25 %, left hippocampus: A=34 % and C=19 %). For volumetric amygdala in both hemispheres, variations were approximately half explained by a combination of genetic and shared environment (right amygdala: A=41 % and C=6 %, left amygdala: A=38 % and C=4 %), even though the variations explained by shared environment are small portions. The variation of right volumetric nucleus accumbens was partly driven by genetic factors (A=38 %) whereas variation of left volumetric nucleus accumbens was partly explained by a combination of genetic and shared environmental input (A=2 % and C=20 %).

*Slope*

Within de affective network, variation in volumetric slopes of the right and left hippocampus was approximately half explained by a combination of genetic control and shared environment (right hippocampus: A=17 % and C=17 %, left hippocampus: A=23 % and C=17 %). Variations in volumetric amygdala (both hemispheres) and right nucleus accumbens slopes were all partly driven by genetic factors (right amygdala: A=22 %, left amygdala=26 %, right nucleus accumbens: A=26 %). Within-twin pair correlations of left nucleus accumbens slopes in MZ and DZ twins were not significant.

#### 3.2.4 Patterns heritability estimates

We conducted a descriptive comparison of heritability estimates between the different regional networks by calculating mean scores of the reported percentages of the contributions of additive genetic (A), common shared environment (C) and unique environment/measurement error (E) for intercept and slope across networks, ROIs, dimensions, and dimensions per network. See Table 4 for an overview of the mean percentages of ACE contributions. **ROIs**

**Table 4:** Mean percentages of ACE contributions in networks, brain regions, dimensions, and dimensions per network.

	**Measure**		***A***	***C***	***E***		***A***	***C***	***E***
**Intercept networks**						**Slope networks**			
	Sensorimotor		33 %	11 %	56 %		16 %	12 %	72 %
	Social		34 %	12 %	54 %		9 %	11 %	80 %
	Affective		30 %	12 %	58 %		23 %	7 %	70 %
**Intercept ROIs**						**Slope ROIs**			
	Sensorimotor	Primary motor	18 %	30 %	52 %		27 %	0 %	73 %
		Somatosensory	35 %	13 %	52 %		17 %	11 %	62 %
		DLPFC	26 %	5 %	69 %		20 %	7 %	83 %
		Cerebellum	59 %	0 %	41 %		2 %	28 %	70 %
	Social	mPFC	33 %	0 %	67 %		22 %	0 %	78 %
		TPJ	25 %	17 %	58 %		1 %	16 %	84 %
		STS	30 %	17 %	53 %		0 %	20 %	80 %
		Precuneus	55 %	10 %	35 %		29 %	0 %	71 %
	Affective	Hippocampus	30 %	22 %	48 %		20 %	17 %	63 %
		Amygdala	40 %	5 %	55 %		24 %	0 %	76 %
		N. accumbens	20 %	10 %	70 %		26 %	0 %	74 %
**Intercept dimensions**						**Slope dimensions**			
		CT	14 %	15 %	71 %		15 %	6 %	79 %
		SA	38 %	14 %	48 %		14 %	11 %	75 %
		VO	37 %	9 %	54 %		17 %	13 %	70 %
**Intercept dimensions per network**						**Slope dimensions per network**			
	Sensorimotor	CT	17 %	12 %	71 %		28 %	0 %	82 %
		SA	32 %	14 %	54 %		17 %	14 %	69 %
		VO	59 %	0 %	41 %		2 %	28 %	70 %
	Social	CT	8 %	20 %	72 %		7 %	10 %	83 %
		SA	53 %	14 %	33 %		1 %	19 %	80 %
		VO	*NA*	*NA*	*NA*		*NA*	*NA*	*NA*
	Affective	CT	*NA*	*NA*	*NA*		*NA*	*NA*	*NA*
		SA	*NA*	*NA*	*NA*		*NA*	*NA*	*NA*
		VO	30 %	12 %	58 %		23 %	7 %	70 %
**Intercept hemisphere**						**Slope hemisphere**			
	Right		35 %	18 %	47 %		17 %	20 %	63 %
	Left		32 %	20 %	48 %		25 %	20 %	55 %

*Note*. ACE contributions: A = additive genetic, C = common shared environment, E = unique environment/measurement error; CT = Cortical thickness, SA = surface area, VO = volume.

As can be seen in Table 4, the descriptive comparison showed differences in heritability estimates between brain regions on the intercepts and slopes within networks, whereas no such difference was shown between networks. The variances of intercepts and slopes of the ROIs were all (except for STS slope) influenced by genetic contribution (intercept A ranging from 18 % to 59 %; slope A ranging from 1 % to 29 %). Also, variances of the intercept and slopes of the ROIs (except for intercepts of cerebellum and mPFC, and for slopes of mPFC, precuneus, amygdala, and nucleus accumbens) were additionally explained by shared environmental factors (intercept C ranging from 5 % to 30 %; slope C ranging from 7 % to 28 %). Furthermore, variances of surface area and volumetric intercepts were to a larger extent explained by genetic factors than cortical thickness (A=38 % surface area versus A=14 % cortical thickness). Surface area and subcortical volumetric development was for a larger extent explained by shared environment compared to cortical thickness (C=11 % surface area versus A=6 % cortical thickness), whereas development of all morphological dimensions was also influenced by genetic contributions (A ranging from 14 % to 17 %). We also computed hemispheric differences in genetic and environmental factors based on intercept and slope. No differences in genetic and environmental effects between hemispheres were observed.

## 4 Discussion

The sensorimotor, social, and affective brain networks are known for their protracted development, extending into early adulthood (Mills et al., 2014, Sanders et al., 2022, Tamnes et al., 2017). We confirmed significant developmental changes from 7 to 14 years of age in 95 % of our ROIs. These developmental patterns may reflect prolonged phases of brain plasticity. Yet, it has not been previously compared to what extent genetical and environmental contributions vary on between subject (i.e., intercept in middle childhood) and within subject variability (i.e., slope between 7 and 14 years old). Furthermore, it is unknown whether genetic and environmental contributions differ per brain region and by morphological brain dimension (i.e., cortical thickness, surface area, and volume). Results showed that regions in middle childhood were all genetically driven (ranging from 18 % to 59 %) but variances in the primary motor cortex (30 %), somatosensory cortex (35 %), DLPFC (5 %), TPJ (17 %), STS (17 %), precuneus (10 %), hippocampus (22 %), amygdala (5 %), and nucleus accumbens (10 %) were additionally explained by shared environment (ranging from 5 % to 30 %). Furthermore, results showed that surface area and volumetric brain structures were to a larger extent driven by genetic factors than cortical thickness (38 % versus 14 %). Next, results showed that longitudinal changes in brain regions within the sensorimotor, social, and affective network were mainly genetically driven (ranging from 1 % to 29 %) but variances in development of the somatosensory cortex (11 %), DLPFC (7 %), cerebellum (28 %), TPJ (16 %), STS (20 %), and hippocampus (17 %) were (additionally) explained by shared environment (ranging from 7 % to 28 %). Surface area and subcortical volumetric development was for a larger extent explained by shared environment compared to cortical thickness, whereas development of all morphological dimensions was also influenced by genetic contributions. Finally, our findings confirmed the absence of differences in genetic and environmental effects between hemispheres.

First, the direction of the trajectories of brain development in the present study align with prior observations in children and early adolescents (Aubert-Broche et al., 2013, Mills et al., 2016, Tamnes et al., 2017, Wierenga et al., 2014, Wierenga et al., 2018). We observed mainly decreases in cortical thickness and increases or stability in cortical surface area and subcortical volume. Furthermore, in line with prior studies and our expectations we observed regional heterogeneity in heritability estimates of brain structure in middle childhood (Jansen et al., 2015, Swagerman et al., 2014, Thompson-Schill et al., 2005, van der Meulen et al., 2020). In contrast, average heritability estimates between networks did not show such differences as the sensorimotor, social, and affective networks are all explained for approximately 33 % by genetic factors, 12 % by shared environment, and 55 % by unique environment/measurement error. More specifically, brain structure in middle childhood of all regions (regardless of morphological dimension) were genetically driven. Additionally, shared environmental effects were observed in a number of regions which was largest for the somatosensory and primary motor cortex of the sensorimotor network, that are involved in receiving sensory input (Raju and Tadi, 2020) and controlling voluntary movements (Sanes and Donoghue, 2000). Prior work argued that these brain regions, which develop phylogenetically and ontologically early, are mediated by genetic input in early childhood (Lenroot et al., 2009; Rosa and Tweedale, 2005). However, they also reported a decrease of genetic contribution with increasing age (between 5 and 18 years old) in these regions (Lenroot et al., 2009). This may imply that environmental influences increase during this specific period. This suggests that there might be a sensitive period for brain plasticity in these motor regions in middle childhood (at age 7) that is driven by environmental input, such as motor-skill learning in sports (Gerver and De Bruin, 2003) or playing an instrument that requires intense practice (Penhune, 2021, van Drunen et al., 2023).

Note that developmental changes of the primary motor cortex were mainly influenced by genetic factors. The discovery that developmental changes in the motor cortex were predominantly influenced by genetic influences, but at age 7, were partly influenced by shared environmental factors, potentially supports the suggestion that middle childhood is a period during which children become increasingly susceptible to environmental influences on controlling motor actions. The cerebellum and DLPFC in middle childhood, involved in motor learning including eye-hand coordination (Miall and Jenkinson, 2005) and selection of action (Hasan et al., 2013), were mainly influenced by genetic factors in line with previous observations in childhood (Lenroot et al., 2009, van et al., 2012). Interestingly, individual differences in cerebellum development were mainly explained by shared environment. This may indicate that the cerebellum, in contrast to the primary motor cortex, is more susceptible for environmentally induced alterations in the period *after* middle childhood which can possibly contribute to the finetuning of motor learning.

Consistent with prior work, there was evidence for shared environmental input on the TPJ and STS of the social network in middle childhood cross-sectionally (van der Meulen et al., 2020). The present study adds to these prior findings by confirming the pattern in a longitudinal sample of a wide age range (7–14 years), and by showing that shared environment also contributed to differences in brain structure of the precuneus in middle childhood. These findings may have implications for studies that related social cognitive processes to social brain regions. Here, TPJ, STS, and precuneus are involved in social cognition, perspective taking, and social decision-making (Blakemore, 2008), which depend on social experiences in the teenage years (Crone and Dahl, 2012, Crone and Fuligni, 2020). Indeed, an earlier study on the same sample but separated for pre- and post-COVID-19 effects already showed that the TPJ showed recovery effects in brain development during childhood after experiencing social restrictions in the COVID-19 pandemic (van Drunen et al., 2023). In the current study, we did not observe shared environmental input on brain structure or development of the mPFC, even though this was observed in a previous study including only the first wave of the current data (van der Meulen et al., 2020). A possible explanation for the lack of shared environmental contributions in our study is that plasticity of the mPFC is age dependent and may become more susceptible for environmental effects when children enter puberty and adolescence. Indeed, prior work showed that increased mPFC thickness and surface area at 14 years of age was associated with friendship quality (Becht et al., 2021). This may suggest a sensitive window for environmental effects on the brain structure of mPFC that takes place after middle childhood and early adolescence. To this end, these differential heritability estimates on brain structure and development in the social network show that subregions in this network are driven by possibly different social contexts that may result in different social behaviors.

We followed upon the results of prior work showing that a combination of genetic and shared environmental factors influence affective brain regions in middle childhood (Brouwer et al., 2017, Swagerman et al., 2014), including mainly age-related increases of subcortical volume between 7 and 14-year-olds. Results in the present study showed that the hippocampus, nucleus accumbens, and amygdala (by a smaller extent) were influenced by genetic factors and were additionally driven by shared environmental input. Moreover, solely longitudinal changes of the hippocampus were explained by shared environment, whereas amygdala and nucleus accumbens development were mostly influenced by genetic contribution. As such, particularly hippocampus structure in middle childhood and development are sensitive for environmental input from childhood to early adolescence compared to the other affective brain regions. The hippocampus is indeed known for its nature of high plasticity (Hanson et al., 2015, Kim and Diamond, 2002). Prior research showed that all these affective brain regions are involved in evaluating emotional significance (Tottenham and Sheridan, 2010), socio-emotional functioning (Kim and Yoon, 1998), and reward learning (Ikemoto and Panksepp, 1999), and thought to be affected by stressful environments (Goff et al., 2013, Kim and Yoon, 1998, Woon and Hedges, 2008). Moreover, our finding showing that a combination of genetic and shared environmental factors influence affective brain regions fits with recent studies showing that hippocampus development (van Drunen et al., 2023) and amygdala cross-sectionally with increasing age (Gotlib et al., 2022) were affected by contextual influences in early adolescence, such as experiencing possible stressors during the COVID-19 pandemic.

Comparison of heritability estimates on morphological dimensions of brain structure in middle childhood showed that surface area and subcortical volume were for a larger extent driven by genetic factors compared to cortical thickness, whereas all dimensions were additionally driven for a similarly small extent by shared environment. These findings are consistent with previous adult studies (Eyler et al., 2012, Panizzon et al., 2009, Strelnikov et al., 2022, Swagerman et al., 2014, Winkler et al., 2010). Moreover, Panizzon et al. (2009) reported that distinct genetic factors influence surface area and cortical thickness, underscoring the need of considering these morphological dimensions separately in genetic modeling which is underlined by our findings. Knowledge on specific genetic contributions that explain individual differences in cortical thickness and surface area would be highly valuable in unraveling biological determinants of complex diseases, such as endophenotypes which are traits that genetically relate to neuropsychiatric disorders (Glahn et al., 2007). For instance, mutations of genes (e.g., GPR56 genes that encode orphan G protein–coupled receptors (GPCR)) in humans have previously been linked to increases in cortical surface area (Piao et al., 2004). These mutations may result in excessive expansions, such as brain cortical malformations, of selected brain regions. Future studies should focus on the exploration of endophenotypes, influencing specifically cortical thickness or surface area, to provide more insight into neuropsychiatric disorders (e.g., schizophrenia or depression) that can already have its onset early in development**.** Note that longitudinal changes in surface area were for a larger extent driven by shared environmental influences, whereas both surface area and cortical thickness were also influenced by genetic contribution. This is in line with prior work on 8–13 year-olds where harsh and inconsistent parenting was associated with accelerated reduction of surface area in medial parietal and temporal pole brain regions (Whittle et al., 2022)**.**

This study had several strengths, it includes a large longitudinal twin MRI dataset (n=485) that allows to investigate heritability estimates on multiple brain regions in a relatively young sample. Furthermore, this study examined genetic and environmental contributions on different morphological dimensions of brain structure and development which are rarely compared within one single study. However, there are limitations that need to be considered when interpreting our findings. The present study mainly focused on the interpretations of additive genetic (A) and common shared environmental (C) contributions, since the unique environmental (E) estimate also incorporates measurement error. As such, additional information on other family members together with classical twin data are needed to distinguish between non-additive genes (D) and unique environment (E) (Keller et al., 2009). Furthermore, it remains unclear which specific genes play a role in the genetic contribution on brain structure and longitudinal brain changes that we observed. For instance, prior work showed that genes (e.g., Brain-Derived Neurotrophic Factor in cerebral cortex and dopamine receptors in prefrontal cortex) can be expressed throughout life and may alter in expression rate during brain maturation processes (Cohen-Cory et al., 2010, Duncan et al., 2010). Future directions are needed to explore the different genetic factors and genetic changes that take place during different phases across the lifespan. Furthermore, based on the availability of three brain measurements, we estimated linear trajectories at an individual level. However, prior work has demonstrated that brain development typically follows more intricate growth patterns (Aubert-Broche et al., 2013, Giedd et al., 1999, Mills et al., 2016, Tamnes et al., 2017, Wierenga et al., 2014). This limitation could be addressed in future studies by incorporating an additional time point for individual levels of brain developmental estimates. Nonetheless, individual differences in developmental change and investigations into associations with these differences can be studied using at least 3 brain measurements (Parsons and McCormick, 2024). Finally, on a group level we observed genetic and environmental influences on brain structure and development, however, we did not identify which individual neurobiological mechanisms account for increased environmental susceptibility (e.g., gene by environment interactions). Nevertheless, this study provides innovative heritability findings using a longitudinal brain sample which can be used as building blocks for future studies investigating brain-behavior associations in different contexts.

Taking together, our results highlight the spatially dependent effects of genetic and environmental influences on brain structure and longitudinal changes in regions of the sensorimotor, social, and affective networks from middle childhood to early adolescence. Furthermore, we showed that surface area was more heritable compared to cortical thickness, whereas longitudinal changes of surface area were increasingly driven by shared environment. These results emphasize the need for further explorations in brain-behavior associations and observe which enriched and deprived contexts are at play in development between childhood and adolescence. Ultimately, this study can contribute to create applicable interventions, such as parenting or teaching programs, that can help children thrive throughout their development.

## Ethics approval

The study and procedures were approved by the Dutch Central Committee on Research Involving Human Subjects (CCMO).

## Author contributions

LvD: data curation; LvD and SD: data investigation; LvD, EAC, and LW: Conceptualization; LvD: Formal analysis, Data curation, Visualization; LvD and LW: Roles/Writing - original draft; LvD, SD, EAC, and LW: Writing - review & editing.

## Declaration of Competing Interest

The authors declare that they have no known competing financial interests or personal relationships that could have appeared to influence the work reported in this paper.

## Data Availability

Data will be made available on request.
