# Supplementary material for: Genetic and environmental influences on structural brain development from childhood to adolescence: A longitudinal twin study on cortical thickness, surface area, and subcortical volume
Source: Dev Cogn Neurosci. 2024 Jun 11;68:101407. doi: 10.1016/j.dcn.2024.101407 (PMC11225697; doi:10.1016/j.dcn.2024.101407)
Supplement: Supplementary file 1 — Supplementary material [file mmc1.docx]

**Supplementary Materials**

Table S1-A Linear mixed model effects of cortical thickness and surface area of ROIs

|  |  |  | **Primary motor** | | **Somatosensory** | | **DLPFC** | | **mPFC** | | **TPJ** | | **STS** | | **Precuneus** | |
| --- | --- | --- | --- | --- | --- | --- | --- | --- | --- | --- | --- | --- | --- | --- | --- | --- |
|  |  |  | **Right** | **Left** | **Right** | **Left** | **Right** | **Left** | **Right** | **Left** | **Right** | **Left** | **Right** | **Left** | **Right** | **Left** |
| **Cortical thickness** |  |  |  |  |  |  |  |  |  |  |  |  |  |  |  |  |
|  | Age | *F* | 28.68 | 11.54 | 8.52 | 9.56 | 4.46 | 36.47 | 186.27 | 206.83 | 28.16 | 22.39 | 23.01 | 32.23 | 162.36 | 135.10 |
|  |  | *P* | **<.001** | **<.001** | **<.01** | **<.01** | <.05 | **<.001** | **<.001** | **<.001** | **<.001** | **<.001** | **<.001** | **<.001** | **<.001** | **<.001** |
|  | Sex | *F* | 2.83 | 2.32 | .04 | .35 | .04 | 4.02 | .39 | 8.83 | 2.79 | 10.07 | 2.93 | 4.28 | 1.33 | .84 |
|  |  | *P* | .09 | .13 | .84 | .55 | .84 | <.05 | .53 | **<.01** | .10 | **<.01** | .08 | <.05 | .25 | .36 |
|  | Zygosity | *F* | 1.35 | 3.06 | .48 | <.01 | .06 | .44 | .21 | 1.09 | .09 | .71 | 1.28 | .91 | 1.88 | 5.39 |
|  |  | *P* | .25 | .08 | .49 | .93 | .81 | .51 | .64 | .30 | .77 | .40 | .26 | .34 | .17 | <.05 |
|  | Age x Sex | *F* | .57 | .90 | .40 | <.001 | .34 | 3.81 | <.01 | .19 | 2.73 | 4.01 | .42 | .80 | 7.62 | 3.22 |
|  |  | *P* | .45 | .34 | .53 | .98 | .56 | .05 | .95 | .67 | .10 | <.05 | .52 | .37 | **<.01** | .07 |
| **Surface area** |  |  |  |  |  |  |  |  |  |  |  |  |  |  |  |  |
|  | Age | *F* | 110.56 | 71.36 | 19.29 | 6.64 | 130.30 | 96.66 | 90.04 | 146.44 | 2.27 | .10 | 80.30 | 27.09 | 37.03 | 33.33 |
|  |  | *P* | **<.001** | **<.001** | **<.001** | **<.05** | **<.001** | **<.001** | **<.001** | **<.001** | .13 | .75 | **<.001** | **<.001** | **<.001** | **<.001** |
|  | Sex | *F* | 66.69 | 68.10 | 36.62 | 35.33 | 52.01 | 53.98 | 12.24 | 21.76 | 24.40 | 44.52 | 45.87 | 54.14 | 52.21 | 60.21 |
|  |  | *P* | **<.001** | **<.001** | **<.001** | **<.001** | **<.001** | **<.001** | **<.001** | **<.001** | **<.001** | **<.001** | **<.001** | **<.001** | **<.001** | **<.001** |
|  | Zygosity | *F* | .06 | 1.27 | 1.04 | 2.93 | .46 | .07 | .66 | .09 | 2.29 | 3.63 | 1.47 | .11 | .33 | .07 |
|  |  | *P* | .81 | .26 | .31 | .09 | .50 | .80 | .42 | .76 | .13 | .06 | .22 | .74 | .56 | .79 |
|  | Age * Sex | *F* | .06 | .02 | .001 | .74 | .08 | .19 | 2.00 | 1.96 | 2.37 | 5.96 | 2.69 | .25 | 1.33 | .01 |
|  |  | *P* | .81 | .64 | .93 | .39 | .78 | .66 | .16 | .16 | .12 | **<.05** | .10 | .62 | .25 | .93 |

Table S1-B Linear mixed model effects of volume of ROIs

|  |  |  | **Cerebellum** | | **Hippocampus** | | **Amygdala** | | **Nucleus Accumbens** | |
| --- | --- | --- | --- | --- | --- | --- | --- | --- | --- | --- |
|  |  |  | **Right** | **Left** | **Right** | **Left** | **Right** | **Left** | **Right** | **Left** |
| **Volume** |  |  |  |  |  |  |  |  |  |  |
|  | Age | *F* | 158.24 | 102.36 | 10.36 | 12.39 | 36.55 | 9.68 | 9.85 | 37.52 |
|  |  | *P* | **<.001** | **<.001** | **<.01** | **<.001** | **<.001** | **<.01** | **<.01** | **<.001** |
|  | Sex | *F* | 31.38 | 29.62 | 45.59 | 36.05 | 46.74 | 51.10 | 14.01 | 9.07 |
|  |  | *P* | **<.001** | **<.001** | **<.001** | **<.001** | **<.001** | **<.01** | **<.001** | **<.01** |
|  | Zygosity | *F* | 2.71 | 2.57 | 1.79 | .65 | .94 | .74 | .01 | 1.63 |
|  |  | *P* | .10 | .11 | .18 | .42 | .33 | .39 | .93 | .20 |
|  | Age * Sex | *F* | 6.43 | 5.91 | .16 | 3.21 | .78 | 8.51 | .04 | .99 |
|  |  | *P* | **<.05** | **<.05** | .69 | .07 | .38 | **<.01** | .84 | .32 |


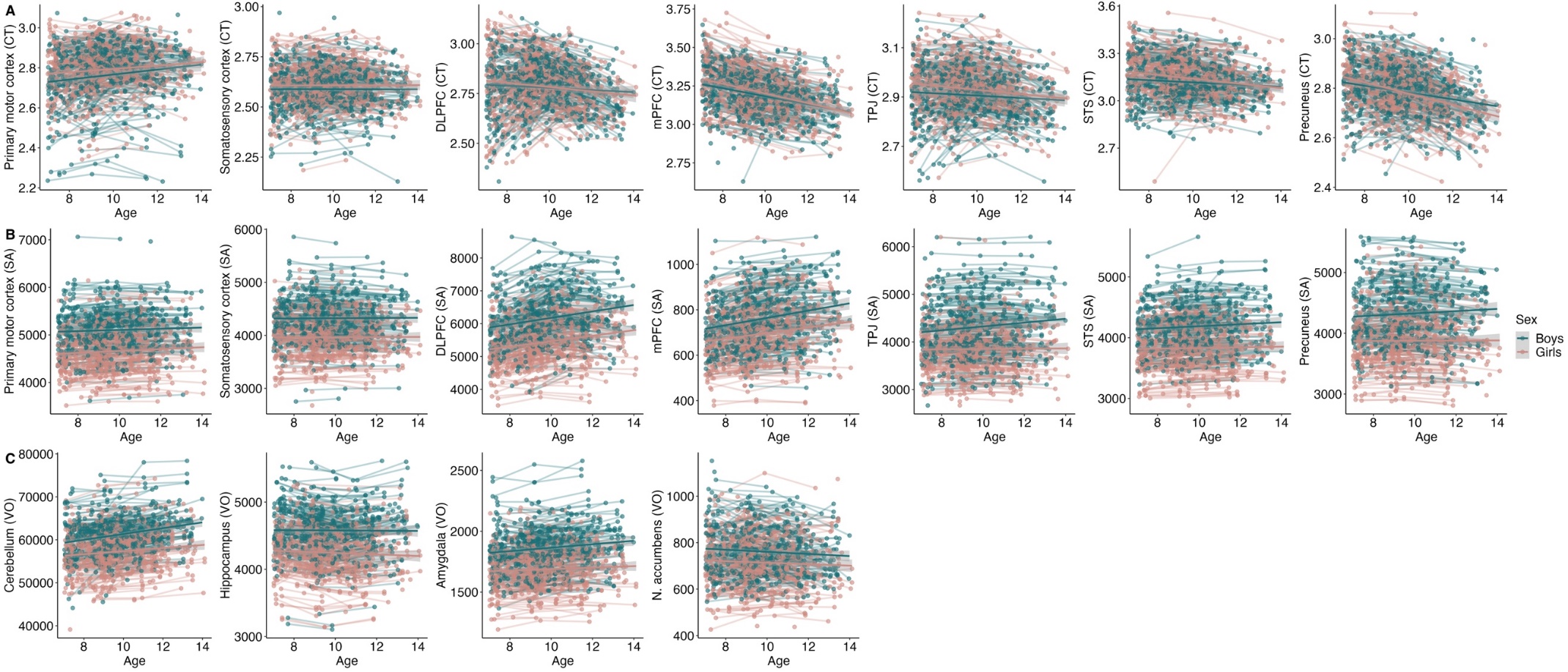


Figure S1. The raw measures of the age-related brain changes of ROIs in the sensorimotor, social, and affective brain networks with A) cortical thickness, B) surface area, and C) subcortical volume. The estimates are based on the linear mixed model results.

Table S2. Within-twin pair correlations and contributions of ACE genetic modeling for intercept (brain structure in early adolescence, age 14).

| **ROI** |  | **MZ** | **DZ** | **Z** | **Model** | **A²** | **C²** | **E²** |
| --- | --- | --- | --- | --- | --- | --- | --- | --- |
| **Sensorimotor network** |  |  |  |  |  |  |  |  |
| Primary motor CT right (i) | *r* | .46 | .15 | 2.73** | *ACE* | **.42** | .00 | .58 |
|  | *p* | <.001 | .19 |  | *95% CI* | **[.00-.56]** | [.00-.31] | [.44-.75] |
| Primary motor CT left (i) | *r* | .16 | .11 | .40 | *ACE* | .11 | .05 | .85 |
|  | *p* | .13 | .32 |  | *95% CI* | [.00-.33] | [.00-.28] | [.67-1.00] |
| Primary motor SA right (i) | *r* | .63 | .45 | 2.02* | *ACE* | **.34** | **.28** | .38 |
|  | *p* | <.001 | <.001 |  | *95% CI* | **[.00-.70]** | **[.00-.59]** | [.28-.52] |
| Primary motor SA left (i) | *r* | .43 | .38 | .47 | *ACE* | .11 | .32 | .57 |
|  | *p* | <.001 | <.01 |  | *95% CI* | [.00-.55] | [0-.52] | [.42-.72] |
| Somatosensory CT right (i) | *r* | .31 | .24 | .60 | *ACE* | .10 | .20 | .70 |
|  | *p* | <.01 | <.05 |  | *95% CI* | [.00-.46] | [.00-.41] | [.54-.87] |
| Somatosensory CT left (i) | *r* | .22 | .04 | 1.45* | *ACE* | **.19** | .00 | .81 |
|  | *p* | <.05 | .71 |  | *95% CI* | **[.00-.37]** | [.00-.27] | [.63-.99] |
| Somatosensory SA right (i) | *r* | .58 | .25 | 3.21** | *ACE* | **.58** | .00 | .42 |
|  | *p* | <.001 | <.05 |  | *95% CI* | **[.24-.70]** | [NA-.27] | [.30-.57] |
| Somatosensory SA left (i) | *r* | .50 | .24 | 2.4** | *ACE* | **.46** | **.01** | .53 |
|  | *p* | <.001 | <.05 |  | *95% CI* | **[.00-.60]** | **[.00-42]** | [.40-.69] |
| DLPFC CT right (i) | *r* | .35 | .20 | 1.28 | *ACE* | .31 | .04 | .65 |
|  | *p* | <.01 | .09 |  | *95% CI* | [.00-.51] | [NA-.39] | [.49-.85] |
| DLPFC CT left (i) | *r* | .39 | .09 | 2.54** | *ACE* | **.36** | .00 | .64 |
|  | *p* | <.001 | .44 |  | *95% CI* | **[.00-.52]** | [.00-.26] | [.48-.82] |
| DLPFC SA right (i) | *r* | .67 | .27 | 4.21*** | *ACE* | **.64** | .00 | .36 |
|  | *p* | <.001 | <.05 |  | *95% CI* | **[.51-.73]** | [NA-.34] | [.27-.49] |
| DLPFC SA left (i) | *r* | .63 | .23 | 3.40*** | *ACE* | **.49** | **.04** | .67 |
|  | *p* | <.001 | <.05 |  | *95% CI* | **[.00-.64]** | **[.00-.47]** | [.35-.61] |
| Cerebellum volume right (i) | *r* | .87 | .40 | 7.17*** | *ACE* | **.86** | .00 | .14 |
|  | *p* | <.001 | <.001 |  | *95% CI* | **[.81-.90]** | [.NA-.24] | [.10-.19] |
| Cerebellum volume left (i) | *r* | .88 | .37 | 7.78*** | *ACE* | **.88** | .00 | .12 |
|  | *p* | <.001 | <.01 |  | *95% CI* | **[.83-.91]** | [NA-.20] | [.09-.17] |
| **Social network** |  |  |  |  |  |  |  |  |
| mPFC CT right (i) | *r* | .21 | .09 | .97 | *ACE* | .22 | .00 | .78 |
|  | *p* | .05 | .45 |  | *95% CI* | [.00-.41] | [.00-.26] | [.56-.99] |
| mPFC CT left (i) | *r* | .18 | .06 | .96 | *ACE* | .18 | .00 | .82 |
|  | *p* | .09 | .64 |  | *95% CI* | [.00-.37] | [.00-.25] | [.63-1.00] |
| mPFC SA right (i) | *r* | .35 | .08 | 2.25* | *ACE* | **.32** | .00 | .68 |
|  | *p* | <.01 | .48 |  | *95% CI* | **[.00-.49]** | [.00-.30] | [.52-.86] |
| mPFC SA left (i) | *r* | .47 | .33 | 1.32 | *ACE* | .27 | .19 | .54 |
|  | *p* | <.001 | <.01 |  | *95% CI* | [.00-.60] | [.00-.50] | [.40-.70] |
| TPJ CT right (i) | *r* | .22 | .29 | -.59 | *ACE* | .02 | .23 | .75 |
|  | *p* | <.05 | <.05 |  | *95% CI* | [.00-.44] | [.00-.38] | [.55-.91] |
| TPJ CT left (i) | *r* | .21 | .20 | .08 | *ACE* | .29 | .00 | .71 |
|  | *p* | <.05 | .09 |  | *95% CI* | [.00-.47] | [.00-30] | [.52-.94] |
| TPJ SA right (i) | *r* | .55 | .25 | 2.86** | *ACE* | **.53** | **.13** | .47 |
|  | *p* | <.001 | <.05 |  | *95% CI* | **[.17-.65]** | **[.00-.29]** | [.35-.63] |
| TPJ SA left (i) | *r* | .29 | .25 | .34 | *ACE* | .18 | .14 | .68 |
|  | *p* | <.01 | <.05 |  | *95% CI* | [NA-.50] | [NA-.40] | [.50-.87] |
| STS CT right (i) | *r* | .32 | .11 | 1.74* | *ACE* | **.31** | **.24** | .69 |
|  | *p* | <.01 | .34 |  | *95% CI* | **[.00-.49]** | **[.00-.26]** | [.51-.89] |
| STS CT left (i) | *r* | .31 | .25 | .51 | *ACE* | .26 | .07 | .67 |
|  | *p* | <.01 | <.05 |  | *95% CI* | [.00-.50] | [.00-.37] | [.50-.88] |
| STS SA right (i) | *r* | .74 | .50 | 3.16** | *ACE* | **.46** | **.27** | .27 |
|  | *p* | <.001 | <.001 |  | *95% CI* | **[.14-.79]** | **[.00-.55]** | [.19-.37] |
| STS SA left (i) | *r* | .61 | .43 | 1.96* | *ACE* | **.29** | **.31** | .40 |
|  | *p* | <.001 | <.001 |  | *95% CI* | **[.00-.68]** | **[.00-.61]** | [.30-.54] |
| Precuneus CT right (i) | *r* | .22 | .04 | 1.45 | *ACE* | .19 | .00 | .81 |
|  | *p* | <.05 | .73 |  | *95% CI* | [.00-.38] | [NA-.24] | [.62-.NA] |
| Precuneus CT left (i) | *r* | .17 | .03 | 1.11 | *ACE* | .15 | .00 | .85 |
|  | *p* | .11 | .83 |  | *95% CI* | [.00-.35] | [.00-.21] | [.65-1.00] |
| Precuneus SA right (i) | *r* | .77 | .41 | 4.61*** | *ACE* | **.72** | **.05** | .23 |
|  | *p* | <.001 | <.001 |  | *95% CI* | **[.38-.83]** | **[.00-.36]** | [.17-.32] |
| Precuneus SA left (i) | *r* | .77 | .36 | 5.07*** | *ACE* | **.78** | .00 | .22 |
|  | *p* | <.001 | <.001 |  | *95% CI* | **[.51-.84]** | [.00-.25] | [.16-.31] |
| Affective network |  |  |  |  |  |  |  |  |
| Hippocampus volume right (i) | *r* | .79 | .32 | 5.83*** | *ACE* | **.78** | .00 | .22 |
|  | *p* | <.001 | <.01 |  | *95% CI* | **[.50-.84]** | [.00-.26] | [.15-.31] |
| Hippocampus volume left (i) | *r* | .73 | .27 | 5.14*** | *ACE* | **.72** | .00 | .28 |
|  | *p* | <.001 | <.01 |  | *95% CI* | **[.46-.79]** | [.00-.23] | [.20-.39] |
| Amygdala volume right (i) | *r* | .73 | .34 | 4.53*** | *ACE* | **.72** | .00 | .28 |
|  | *p* | <.001 | <.01 |  | *95% CI* | **[.36-.80]** | [.00-.33] | [.20-.38] |
| Amygdala volume left (i) | *r* | .74 | .38 | 4.34*** | *ACE* | **.63** | **.09** | .28 |
|  | *p* | <.001 | <.01 |  | *95% CI* | **[.28-.79]** | **[.00-.42]** | [.20-.39] |
| N. accumbens volume right (i) | *r* | .40 | .36 | .37 | *ACE* | .00 | .38 | .62 |
|  | *p* | <.001 | <.01 |  | *95% CI* | [.00-.48] | [.00-.50] | [.48-.76] |
| N. accumbens volume left (i) | *r* | .29 | -.01 | 3.14** | *ACE* | **.20** | .00 | .80 |
|  | *p* | <.01 | .40 |  | *95% CI* | **[.00-.37]** | [.00-.21] | [.63-.98] |

*Note*. i = intercept; mPFC = medial prefrontal cortex; STS = superior temporal sulcus; TPJ = temporoparietal junction; DLPFC = dorsolateral prefrontal cortex; r = Pearson correlation; Z = test statistic z, significant Z-scores indicate significant difference between MZ and DZ correlations. **p*<.05, ***p*<.01, ****p*<.001.
